# Corneal biomechanical characteristics following small incision lenticule extraction for myopia and astigmatism with 3 different cap thicknesses

**DOI:** 10.1186/s12886-023-02786-8

**Published:** 2023-01-30

**Authors:** Xiaotong Lv, Fengju Zhang, Yanzheng Song, Changbin Zhai, Ning Guo, Lingbo Lai, Yushan Xu

**Affiliations:** Beijing Tongren Eye Center, Beijing Tongren Hospital, Capital Medical University, Beijing Ophthalmology & Visual Sciences Key Lab, No 1, Dongjiaominxiang Street, Dongcheng District, Beijing, 100730 China

**Keywords:** Refractive surgery, SMILE, Cap thickness, Myopia, Biomechanics

## Abstract

**Background:**

The design of cap thickness for small incision lenticule extraction (SMILE) plays a role in post-laser vision correction (post-LVC) corneal biomechanics. This study aimed to compare the corneal biomechanical characteristics following SMILE with different cap thicknesses of 110 μm, 120 μm, and 130 μm for myopia and myopic astigmatism correction.

**Methods:**

Seventy-five patients (146 eyes) who underwent SMILE with designed cap thickness of 110 μm, 120 μm, and 130 μm were recruited at the Eye Center of Beijing Tongren Hospital between August 2020 and November 2021. Visual acuity, refraction, and corneal biomechanical parameters were measured preoperatively, 1 week and 1, 3, 6 months postoperatively. One-way analysis of variances (ANOVA) with Bonferroni correction or Kruskal-Wallis test was performed to compare the parameters among different groups. Repeated-measures analysis of variance with Bonferroni correction or Friedman test was applied for comparing the parameters within different follow-up times.

**Results:**

Uncorrected distance visual acuity of 110-μm group was better only at 1-week and 1-month postoperatively (*P* = 0.012, 0.037). There were no significant differences in spherical equivalent, nor in Corvis biomechanical index-laser vision correction (CBI-LVC). All the parameters reached stability at 3-month postoperatively. Integrated radius (IR) and deformation amplitude ratio 2 mm (DA ratio 2 mm) in 120-μm and 130-μm groups were higher than 110-μm group at 1-month postoperatively (*P* = 0.019, 0.002). So was Ambrósio relational thickness (ARTh) at 6-month postoperatively (*P* = 0.011). Stiffness parameter at applanation A1 (SP-A1), stress-strain index (SSI), biomechanically corrected intraocular pressure (bIOP) and central corneal thickness (CCT) were highest in 130-μm group, followed by 120-μm group, then 110-μm group at 3-month (*P*<0.001, *P* = 0.030, *P* = 0.027, *P* = 0.008) and 6-month (*P*<0.001, *P* = 0.002, *P* = 0.0023, *P* = 0.001) postoperatively.

**Conclusions:**

The corneal stiffness following SMILE was greatest with 130-μm cap, followed by 120-μm cap, then 110-μm cap. 130-μm cap might have advantages in terms of corneal biomechanics and retreatment option. The SMILE-designed protocol should be customized in practice.

**Supplementary Information:**

The online version contains supplementary material available at 10.1186/s12886-023-02786-8.

## Background

Nowadays, laser refractive surgery has become one of the most prevalent procedures to correct refractive error for adults with efficacy and safety [1]. With the introduction of femtosecond laser to refractive surgery, the small incision lenticule extraction (SMILE) was developed, which is an advanced procedure with micro-incision and without corneal flap [2, 3]. As the surgery is conducted mainly through ablation on cornea, the structure and shape of cornea are altered simultaneously, which may potentially lead to certain biomechanical properties changes [4]. Corneal biomechanics represents the ability of resistance against extraocular and intraocular pressure and plays an important role in the safety as well as stability of refractive surgery [5, 6]. Based on the various structure and organization of collagen fibrils as well as lamellae, the corneal biomechanics is spatially inhomogeneous and the anterior corneal stroma is stiffer than the posterior stroma [7].

It is suggested that different designs of cap thickness in SMILE procedure may lead to different post-laser vision correction (post-LVC) corneal biomechanics [8]. On one hand, a thicker cap means less disruption of the anterior stromal, probably a positive factor for post-LVC biomechanics. But on the other hand, a thicker cap means a thinner residual stroma, which is a negative factor for post-LVC biomechanics [9]. These two factors make the biomechanical changes confusing theoretically. The range of the corneal cap thickness is 100–160 μm [2, 10] while 110 μm, 120 μm and 130 μm are clinically more prevalent in China. Although several studies have investigated the biomechanical outcome after SMILE with different caps [11–16], either the measurements or the parameters were varied, and no studies have simultaneously compared the differences among 110 μm, 120 μm and 130 μm cap.

Furthermore, the cap thickness of the primary SMILE determines the retreatment option when refractive regression occurs [17]. Surface ablation and CIRCLE (cap-to-flap) procedure are the common enhancement methods for SMILE while the thin-flap LASIK is seldom used due to the increased risk of buttonholes [18]. As the visual outcomes of surface ablation and CIRCLE enhancement after SMILE were comparable, surface ablation has a biomechanics advantage over CIRCLE due to fewer collagen lamellae being cut off, surface ablation becomes an excellent option for enhancement [19]. A thinner initial corneal cap might result in the opening of the cap because a thin cap may not be enough to afford the ablation depth, which means the patients have to take a risk of complications and biomechanical weakening related to the flap. Therefore, the cap thickness plays a crucial role in SMILE.

This prospective study was designed to investigate and compare the corneal biomechanics assessed by Corvis ST (Oculus Optikgeräte GmbH, Wetzlar, Germany) and the new software (version 1.6r2031) following SMILE with different cap thicknesses of 110 μm, 120 μm, and 130 μm for myopia and myopic astigmatism correction, so as to appraise the clinical importance of these cap thicknesses further.

## Methods

### Patients

A prospective case-control study. 75 patients (146 eyes) were recruited at the Eye Center of Beijing Tongren Hospital between August 2020 and November 2021. The inclusion criteria were 18 years or older, the central corneal thickness (CCT) between 510 μm and 580 μm bilaterally, the manifest refraction spherical equivalent (MRSE) between − 3.00 diopters (D) and − 8.00D, stable refraction (alteration of 1.00D or less) for at least 2 years, corrected distance visual acuity (CDVA) of 20/25 or better, refrained from soft contact lenses for at least 2 weeks and rigid contact lenses for at least 4 weeks and orthokeratology lenses for at least 12 weeks before surgery. The exclusion criteria included calculated residual stromal thickness less than 280 μm, presence or history of ocular and systemic diseases, or history of ocular surgery. This study was conducted in compliance with the Declaration of Helsinki and was approved by the institutional review board of Beijing Tongren Hospital. Written informed consent was obtained from all patients before surgery.

### Measurements

All the participants underwent a thorough ophthalmologic examination for both eyes preoperatively, including uncorrected distance visual acuity (UDVA), CDVA [logarithm of the minimum angle of resolution (logMAR) scale], manifest refraction, intraocular pressure by non-contact tonometer, slit lamp and fundus examination. The Corvis ST (Oculus Optikgeräte GmbH, Wetzlar, Germany) measurement was performed by the same experienced physician (XTL) and carried out between 09.00 am and 04.00 pm [20]. Before each measurement, the patients were asked to blink eyes to achieve regular tear film. Only the examinations with a quality score of ‘OK’ were included for analysis. The biomechanical parameters were recorded and obtained. UDVA, slit lamp examination, refraction, along with the Corvis ST were acquired preoperatively, at 1 week and 1, 3, 6 months postoperatively.

### Surgical technique

After analysis of the preoperative clinical files, the cap thickness was designed by the same experienced surgeon (FJZ) as 110 μm, 120 μm, and 130 μm for SMILE treatment. Preoperatively, all patients received antibiotic eye drops for 3 days. All the surgeries were performed by the same surgeon (FZ) using the Visumax femtosecond laser system (Carl Zeiss Meditec AG, Jena, Germany) with a repetition rate of 500 kHz and pulse energy of 130 nJ. The cap thickness of the bilateral eyes of each patient was the same and the optical zone was 6.5 mm. The 2-mm incision was created at the 12 o’clock position. Antibiotic, steroid and artificial tear drops were administered for 2–4 weeks at post-op.

### Statistical analysis

The Shapiro–Wilk test was applied for testing the normality of data. Normally distributed data were described as mean ± standard deviation, and were analyzed by the one-way analysis of variances (ANOVA) with Bonferroni correction. Repeated-measures analysis of variance with Bonferroni correction (the preoperative value as a covariate) was used for comparing the parameters within different follow-up times. Non-normally distributed data were presented as median and quartile ranges and were analyzed by the Kruskal-Wallis test or Friedman test. P<0.05 was considered statistically significant. The statistical analysis was performed by SPSS software (version 26.0; IBM, Chicago, IL, USA).

## Results

146 eyes of 75 patients were included in this study. There were 28 men and 47 women with a mean age of 25.0 ± 4.0 (18 to 33) years. 16 patients were not followed up at 6 months postoperatively. The demographic and basic characteristics of the three groups are summarized in Table 1 There were no statistically significant differences among the three groups other than residual stromal bed thickness. The calculated RST was thicker with the thinner cap in sequence (*P*<0.001).

**Table 1:** Demographic and basic characteristics of patients

Parameter	110 μm	120 μm	130 μm	***P*** Value
Eyes, n	48	49	49	N/A
Female, n	13 (52%)	14 (56%)	12 (48%)	0.852
Right eye, n	25 (52%)	24 (49%)	25 (51%)	0.953
Age, y	24.04 ± 4.42	25.47 ± 4.36	24.02 ± 4.08	0.163
CDVA (LogMAR)	−0.07 ± 0.02	−0.06 ± 0.03	−0.06 ± 0.03	0.065
MRSE (D)	−5.29 ± 0.97	−5.26 ± 0.91	−4.98 ± 0.84	0.172
S (D)	−5.02 ± 0.99	−4.87 ± 1.01	−4.60 ± 0.82	0.094
C (D)	−0.55 ± 0.51	−0.79 ± 0.69	−0.75 ± 0.81	0.190
Ks (D)	44.00 ± 1.07	44.44 ± 1.24	44.07 ± 1.21	0.170
Kf (D)	42.90 ± 1.16	43.12 ± 0.97	42.81 ± 1.36	0.432
CCT (μm)	545.25 ± 15.02	546.59 ± 14.98	551.33 ± 9.89	0.069
LT (μm)	116.08 ± 14.55	118.94 ± 13.78	114.76 ± 15.08	0.347
RST (μm)	319.17 ± 15.63	308.12 ± 12.62	306.98 ± 11.07	< 0.001*

*CDVA* Corrected distance visual acuity, *LogMAR* Logarithm of the minimum angle of resolution, *MRSE* Manifest refraction spherical equivalent, *S* Spherical error, *C* Cylindrical error, *D* Diopter, *N/A* Not applicable, *Ks* Steep keratometry, *Kf* Flat keratometry, *CCT* Central corneal thickness, *LT* Lenticule thickness, *RST* Residual stromal bed thickness

*P < 0.05

All the surgeries were completed successfully without complications. No postoperative complications occurred during the follow-up, and no cases of retreatment were found until December 2022. Table 2 displays the postoperative outcomes of visual acuity and refraction error. The mean UDVA (LogMAR) of the 110-μm group (1 week: − 0.12 ± 0.17; 1 month: − 0.09 ± 0.06) was better than that of the 120-μm group (1 week: − 0.05 ± 0.08; 1 month: − 0.06 ± 0.06) at 1-week and 1-month postoperatively (*P* = 0.009, 0.018). There were no statistically significant differences of the mean UDVA among the three groups at other time points after SMILE (*P*>0.05). As for the mean spherical equivalent, no statistically significant differences were observed in different cap thicknesses groups at either follow-up time point (*P*<0.05).

**Table 2:** Postoperative outcomes of visual acuity and refraction errors

Parameter	Time	110 μm	120 μm	130 μm	***P*** Value
UDVA (LogMAR)	1 w	−0.12 ± 0.17	−0.05 ± 0.08	−0.07 ± 0.06	0.012*
	1 m	−0.09 ± 0.06	−0.06 ± 0.06	−0.08 ± 0.06	0.037*
	3 m	−0.08 ± 0.07	−0.11 ± 0.07	−0.10 ± 0.07	0.180
	6 m	−0.11 ± 0.06	−0.10 ± 0.07	−0.09 ± 0.06	0.452
SE (D)	1 w	−0.06 ± 0.55	−0.12 ± 0.40	0.07 ± 0.39	0.114
	1 m	0.09 ± 0.42	0.02 ± 0.55	0.16 ± 0.31	0.292
	3 m	0.19 ± 0.42	0.08 ± 0.38	0.17 ± 0.29	0.302
	6 m	0.07 ± 0.34	0.09 ± 0.31	0.09 ± 0.21	0.970

*UDVA* Uncorrected distance visual acuity, *LogMAR* Logarithm of the minimum angle of resolution, *SE* Spherical equivalent, *D* Diopter, *1w* 1-week postoperation, *1 m* 1-month postoperation, *3 m* 3-month postoperation, *6 m* 6-month postoperation

*P < 0.05

**Fig. 1:**
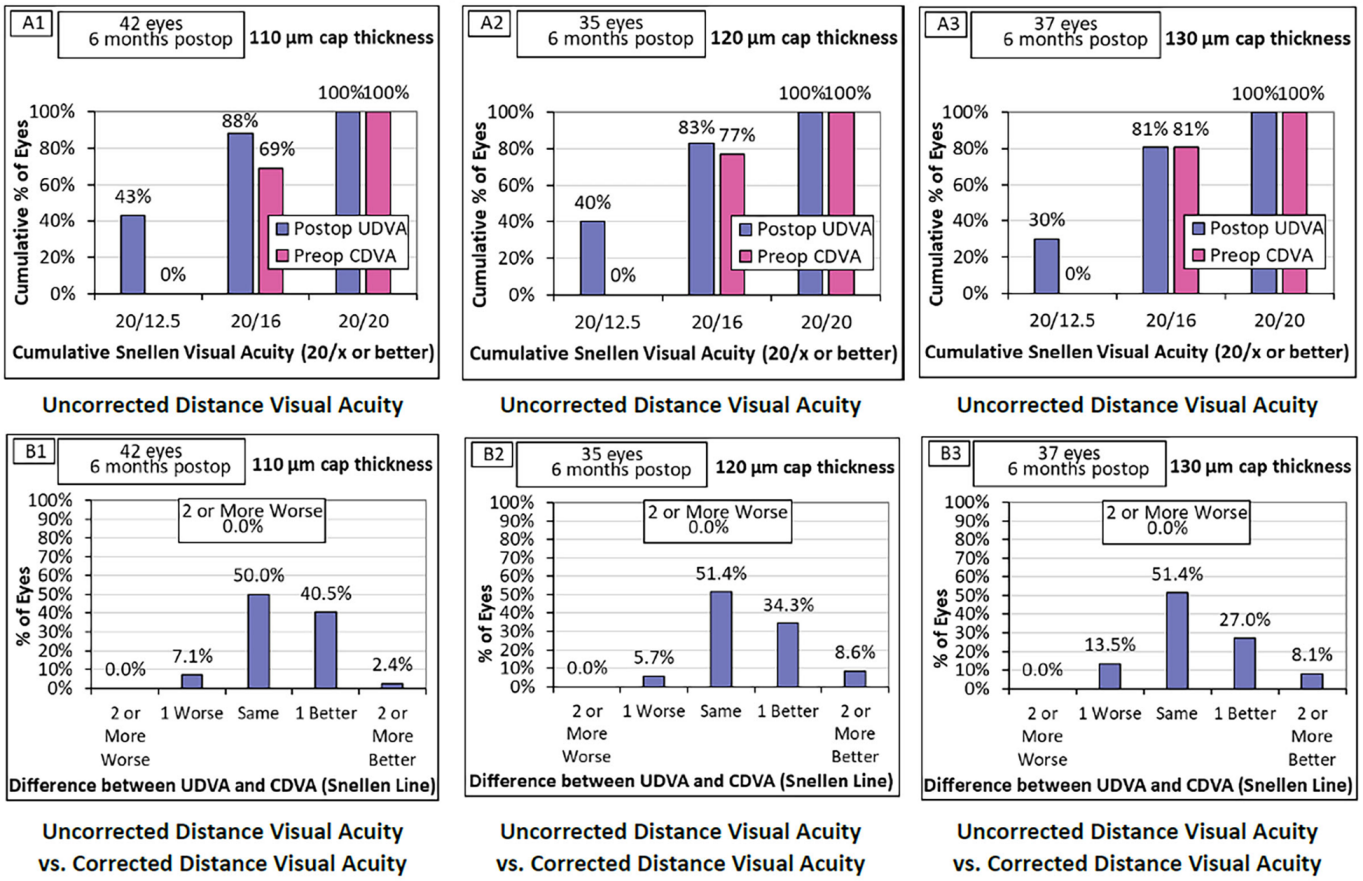
Graphs for comparing the 6-month postoperative visual acuity outcomes of the SMILE with different cap thicknesses. **A** Comparison of the cumulative percentage of eyes for Snellen visual acuity between 6-month postoperative UDVA (uncorrected distance visual acuity) and preoperative CDVA (corrected distance visual acuity). All the eyes achieved UDVA (Snellen) of 20/20 or better at the 6-month follow-up. **B** Difference of the Snellen line between 6-month postoperative UDVA and preoperative CDVA. None of the eyes lost UDVA more than 2 lines versus the preoperative CDVA. The eyes which lost 1 line of Snellen all had the preoperative CDVA of 20/16 and the postoperative UDVA of 20/20

All the patients achieved UDVA (Snellen) of 20/20 or better at the 6-month follow-up, and no patient lost UDVA more than 2 lines versus the preoperative CDVA as shown in Fig.1 The cumulative percentage of eyes with postoperative UDVA at 20/16 or better in 110-μm, 120-μm, and 130-μm group was 88% (37/42), 83% (29/35), and 81% (30/37) (*P* = 0.671) versus preoperative CDVA was 69% (29/42), 77% (27/35), and 81% (30/37) (*P* = 0.446). The cumulative percentage of eyes with postoperative UDVA at 20/12.5 or better in 110-μm, 120-μm, and 130-μm group was 43% (18/42), 40% (14/35), and 30% (11/37) (*P* = 0.460). As for the change of postoperative UDVA and preoperative CDVA in 110-μm, 120-μm, and 130-μm group, there were 7.1% (3/42), 5.7% (2/35), 13.5% (5/37) of eyes lost 1 line of Snellen (*P* = 0.469), all these 10 eyes had the preoperative CDVA of 20/16 and the postoperative UDVA of 20/20. 50.0% (21/42), 51.4% (18/35), 51.4% (19/37) of eyes achieved the same line of Snellen (*P* = 0.990). 40.5% (17/42), 34.3% (12/35), 27.0% (10/37) of eyes gained 1 line of Snellen (*P* = 0.454). 2.4% (1/42), 8.6% (3/35), 8.1% (3/37) of eyes gained 2 or more lines of Snellen (*P* = 0.441). None of the differences were statistically significant.

**Fig. 2:**
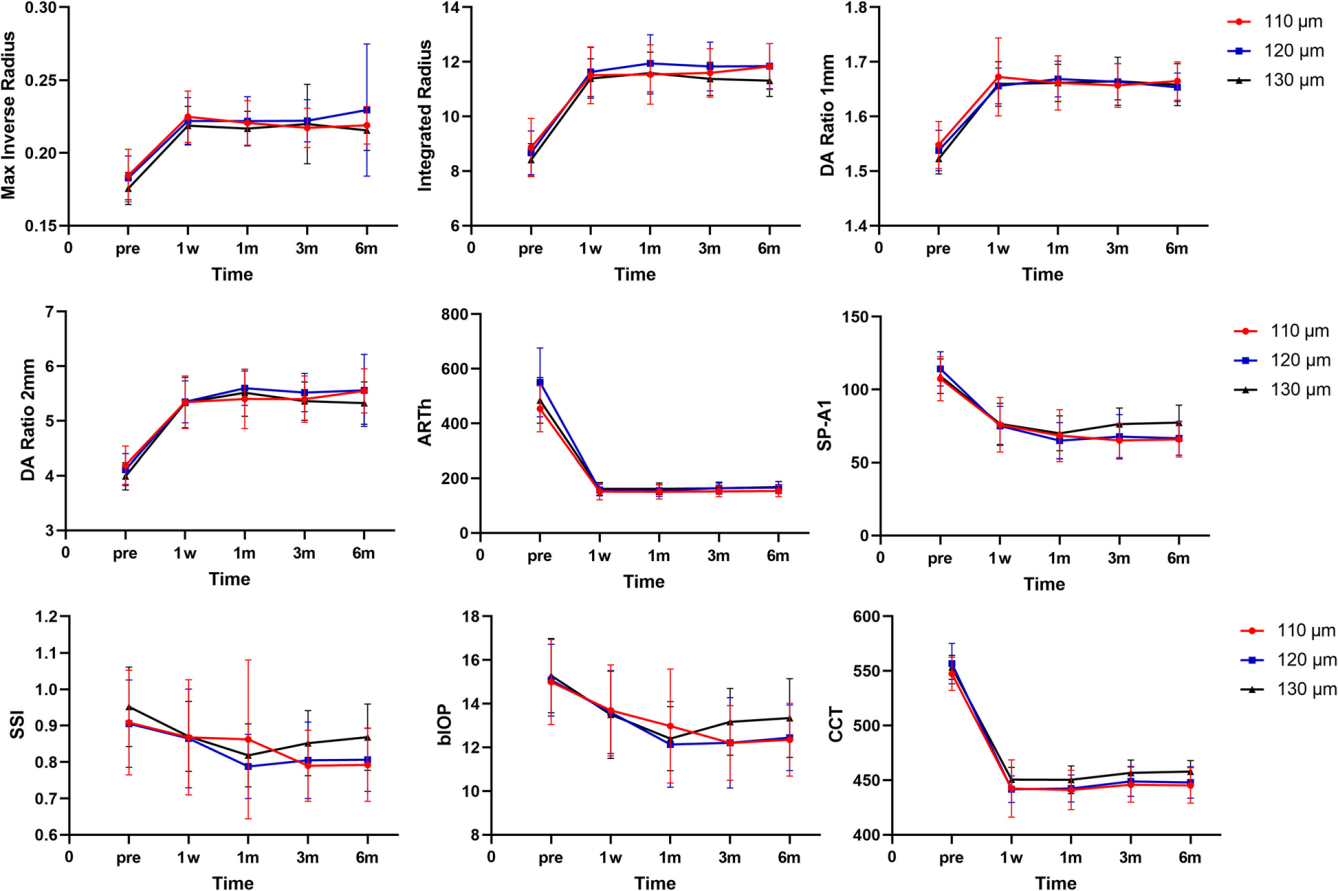
Characteristics of corneal biomechanical parameters following SMILE. Pre represents preoperation; 1w, 1-week postoperation; 1 m, 1-month postoperation; 3 m, 3-month postoperation; 6 m, 6-month postoperation

The corneal biomechanical parameters max inverse radius (M), integrated radius (IR), and deformation amplitude ratio (DA ratio) significantly increased after SMILE in all three groups (*P*<0.001). Additionally, Ambrósio relational thickness (ARTh), stiffness parameter at applanation A1 (SP-A1), stress-strain index (SSI), biomechanically corrected intraocular pressure (bIOP) and central corneal thickness (CCT) all decreased postoperatively (*P*<0.001) as revealed in Fig.2 It was shown that all the parameters above reached stability at 3-month postoperatively in three groups (*P*>0.05).

Considering the difference of the preoperative biomechanics among groups, the preoperative value was utilized as a covariate to reduce error variance and insure statistical balance. There were no statistically significant differences of M or DA ratio 1 mm in either group at any time point after SMILE (*P*>0.05). At 1-month postoperatively, the IR and DA ratio 2 mm in 120-μm and 130-μm groups were both significantly higher than in 110-μm group (*P* = 0.019, 0.002) while no significant differences were found at other time points. The ARTh was significantly higher in 120-μm and 130-μm groups than in 110-μm group only at 6-month postoperatively (*P* = 0.011). In terms of SP-A1, SSI, bIOP, and CCT, they were all significantly higher in a thicker cap group (130-μm group, 120-μm group, and 110-μm group, respectively) at 3-month and 6-month postoperatively (3-month: *P*<0.001, *P* = 0.030, *P* = 0.027, *P* = 0.008; 6-month: *P*<0.001, *P* = 0.002, *P* = 0.023, *P* = 0.001) while none of the differences among groups were significant at other follow-ups. Moreover, the Corvis biomechanical index-laser vision correction (CBI-LVC) showed no significant differences neither in different groups (*P* = 0.590, 0.244, 0.990, 0.096) nor at different follow-up points postoperatively (*P* = 0.790, 0.679, 0.195). An additional movie file shows these in more detail (Additional file 1).

## Discussion

The most commonly applied cap thicknesses of SMILE are 110 μm, 120 μm and 130 μm in China at present. It’s still confusing for surgeons which thickness of cap could result in better clinical outcomes. Despite studies have been conducted to investigate the biomechanical outcome after SMILE with different cap thicknesses such as 140 μm, 150 μm and 160 μm which are not applied in clinic commonly, the simultaneous comparison and dynamic observation of the corneal biomechanics among cap thicknesses of 110 μm, 120 μm and 130 μm are not available so far.

This study investigated the dynamic variations and outcomes of corneal biomechanics as well as visual acuity after SMILE with cap thickness of 110 μm, 120 μm, and 130 μm to shed more light on the better choice for clinical practice. The Corvis ST was applied for more comprehensive analysis, especially a series of new biomechanical parameters derived from it. We found that the corneal biomechanics measured after SMILE with thicker cap was greater than that of thinner cap (110 μm, 120 μm versus 130 μm), corresponding to the clinical study of Wu et al. (110-μm versus 130-μm cap) which only assessed 3 Corvis ST parameters [14] and in vitro study performed by Damgaard et al. (110-μm versus 160-μm cap) which applied inflation test [12].

However, it was displayed that the corneal biomechanics was greater in thinner cap group than thicker cap group (120-μm versus 140-μm) with the nomogram in which the lenticule thickness of SMILE with 120-μm cap was lower than 140-μm cap [16], and a similar result was reported between 110-μm and 140-μm cap [15]. The lenticule thickness plays an essential role in the change of corneal biomechanics post-SMILE and was comparable in our study. We consequently assume that the discrepant biomechanical outcomes were probably attributed to the difference of ablation above. Additionally, the consumption of residual stromal thickness imposed by the 140-μm cap design might be the critical factor for the significant weakening of corneal biomechanics, which is also the reason for the limited application of 140-μm cap in practice. Our results embodied the biomechanical advantage of a thicker cap (110-μm, 120-μm versus 130-μm cap) for SMILE when the ablation condition was semblable.

In this study, we found SP-A1, SSI, bIOP, and CCT were highest in 130-μm cap group, then 120-μm group, and lowest in 110-μm group at 3-month and 6-month postoperatively. ARTh in 120-μm and 130-μm groups were higher than 110-μm group only at 6-month postoperatively. It is indicated that the corneal resistance to deformation was highest to lowest postoperatively in the sequence of 130-μm, 120-μm and 110-μm cap thickness applied in SMILE, demonstrating that the 130-μm cap could preserve the stiffness of cornea more preferably than a thinner cap in this study.

In the respect of other parameters, integrated radius (IR) and DA ratio 2 mm in 120-μm and 130-μm groups were both higher than 110-μm group only at 1-month postoperatively whereas no significant difference was found in groups since 3-month postoperatively. As all the measured corneal biomechanical parameters reached stability at 3-month postoperatively, it is illustrated the deformation of cornea after SMILE with 120-μm and 130-μm cap was transiently greater than that with 110-μm cap at the early stage.

To our knowledge, this is the first prospective study that compares the new parameter CBI-LVC after SMILE applicating different cap thicknesses until now. CBI-LVC was shown to be of high sensitivity and specificity (sensitivity of 93.3% and specificity of 97.8% with a cut-off value of 0.2) for diagnosing post-LVC ectasia by distinguishing the stable corneas from the pathological ones [21], as well as good repeatability after SMILE [22]. Compared to CBI, CBI-LVC is targeted at patients after corneal refractive surgery and is not affected by corneal thickness, thus breaking through the obstacle of CBI in corneal biomechanical evaluation after laser corneal refractive surgery. At 6-month postoperatively, all the CBI-LVC in the present study were lower than the diagnostic cut-off value of 0.2, which implicated the stable post-SMILE condition of this biomechanical index. Furthermore, the CBI-LVC of the three groups was similar during the 6-month follow-up, informing that the biomechanical property for the respect of this index was all maintained at a safe range after incision at 110-μm, 120-μm, and 130-μm depth during SMILE in the current study.

The algorithm SSI was developed to supply an estimate of the material stress-strain behavior in healthy cornea, which quantifies the corneal tissue’s mechanical stiffness without relevance to CCT or bIOP [23]. Higher value of SSI is indicative of higher tissue stiffness. On the other hand, for estimating the stiffness of the whole cornea, the SP-A1 is defined as resultant pressure divided by displacement and has strong correlations to both pachymetry and DA Ratio 2 mm. It was exhibited that the stiffer eyes with greater SP-A1 had higher magnitude pachymetry and bIOP along with lower magnitude DA Ratio 2 mm [24], and so did our results coincide. Therefore, it is suggested that not only the material stiffness of cornea but also the whole stiffness was higher in the 130-μm cap group than 120-μm and 110-μm cap group in this study.

Despite no significant differences observed in preoperative CCT and lenticule thickness, the postoperative CCT was analyzed with a significant difference at 3-month and 6-month postoperatively. Luft et al. [25] detected that the central stroma thickness measured by SD-OCT (spectral-domain optical coherence tomography) kept increasing from 6 weeks to 1 year after SMILE, and perceived it as a manifestation of stromal remodeling following the disruption to collagen lamellae executed by the surgery. We hypothesize that the various stromal remodeling after intra-lamellar incision at different depths of stroma might be the reason for the phenomenon in this study [26, 27], which needs further research. The ARTh was generated for the corneal thickness profile and progression [28]. The high value of ARTh means that the cornea is thick and the thickness slowly increases from the center to the periphery. For the present study, the difference of postoperative ARTh represented the different thickness distribution characteristic of cornea post-SMILE among groups that the corneal thickness was greater and progressed more slowly after SMILE with 120-μm and 130-μm cap than 110-μm cap, which was consistent with the result of postoperative CCT.

Concerning the visual acuity, it was shown that all the patients ultimately maintained UDVA (Snellen) of 20/20 or better and none of the patients lost UDVA more than 2 lines versus the preoperative CDVA at 6-month postoperatively, which confirms the safety and efficacy of SMILE compatible with previous studies [29]. In terms of cap thickness, the cumulative percentage of eyes with postoperative UDVA at 20/16 or better was highest in 110-μm group, then 120-μm group, and 130-μm group for the last, so was the UDVA at 20/12.5 or better. Yet all the differences did not reach statistical significance or clinical relevance. Likewise, the three groups had various but statistically nonsignificant proportions of the postoperative UDVA relative to the preoperative CDVA. Besides, no eyes lost 2 or more lines of Snellen, the eyes which lost 1 line of Snellen all had the preoperative CDVA of 20/16 and the postoperative UDVA of 20/20. It is perceived that 1-line worse UDVA in this analysis method is within normal biological variability [30]. Hence the safety and efficacy of SMILE were similar in the three groups.

Furthermore, the UDVA (LogMAR) in the 110-μm group was better than the thicker cap groups at the early postoperative stage without significant differences of SE. It was supposed by Liu et al. [13] that the easier corneal shape modification might account for the better visual quality of 110-μm cap group at the very early stage after SMILE. However, there were no significant differences of visual acuity when the curvature of the anterior surface differed from each other [15]. The study of Weng et al. [31] showed that the stromal surface created by the thinner caps were smoother than that created by the thicker caps after SMILE, in which the qualitative regularity was analyzed using the scanning electron microscopy. And it has been demonstrated that the smoother stromal surface was associated with better visual outcomes [32]. Therefore, we speculate that the regularity of the stromal surface after SMILE with 110-μm cap was better, which might be associated with the better UDVA in the 110-μm group theoretically. In addition, no significant differences of UDVA were observed since 3-month post-SMILE, demonstrating the comparable visual acuity outcome in the 110-μm, 120-μm, and 130-μm group.

According to our findings, the SMILE designed with 130-μm cap ultimately resulted in greater postoperative corneal stiffness and strength than 120-μm and110-μm cap, indicating the 130-μm cap design had its biomechanical advantage in SMILE. Meanwhile, we found the 6-month postoperative visual acuity was comparable in SMILE using 110-μm, 120-μm, and 130-μm cap. Moreover, the thicker cap could offer an opportunity for surface ablation for enhancement post-SMILE if refractive regression occurs, rather than CIRCLE, so that avoiding the risk of complications related to the flap and benefitting in less biomechanical alteration [19]. It is illustrated that the 130-μm cap has advantages over the 120-μm and 110-μm cap in terms of corneal biomechanics and retreatment option. Even though, the residual stromal bed should never be neglected because it plays an important role in the factors associated with iatrogenic ectasia post-LVC [9, 33]. All the RST of our study was greater than 280 μm (total over 400 μm including cap), within the safe range. Since a thicker cap was accompanied by a thinner RST, it is essential for surgeons to consider all the clinical conditions, such as refractive error, corneal curvature, preoperative CCT, corneal cap, and RST, in order to formulate an adequate surgery and produce a satisfactory result.

The limitations of this study should be mentioned. The ultimately followed-up sample size is relatively small. Besides, longer-term results are warranted to further monitor the stability of the corneal biomechanical property and the condition of iatrogenic ectasia, along with the clinical relevance of CBI-LVC. To address a more comprehensive understanding of the post-SMILE outcomes influenced by cap thickness, it is promising to investigate the corneal remodeling post-SMILE with different cap thicknesses through micro-methods, for instance, confocal microscopy assessment in vivo.

## Conclusion

In conclusion, the SMILE designed with cap thicknesses of 110 μm, 120 μm, and 130 μm were all safe and effective for 6-month follow-up in this study. Corneal biomechanics following SMILE reached stable since 3-month postoperatively and was greatest with 130-μm cap, followed by 120-μm cap, then 110-μm cap. The 130-μm cap might have advantages in terms of corneal biomechanics and retreatment option. In practice, the SMILE-designed protocol should be customized based on the synthetical evaluation of condition.

## Supplementary Information

**Additional file 1.** Corneal Biomechanical Parameters at Different Time Points in Three Groups.

## Data Availability

The datasets used and/or analysed during the current study are available from the corresponding author on reasonable request.

## Abbreviations

SMILE: Small incision lenticule extraction
Post-LVC: Post-laser vision correction
M: Max inverse radius
IR: Integrated radius
DA ratio: Deformation amplitude ratio
ARTh: Ambrósio relational thickness
SP-A1: Stiffness parameter at applanation A1
SSI: Stress-strain index
bIOP: Biomechanically corrected intraocular pressure
CBI-LVC: Corvis biomechanical index-laser vision correction
UDVA: Uncorrected distance visual acuity
CDVA: Corrected distance visual acuity
LogMAR: Logarithm of the minimum angle of resolution
MRSE: Manifest refraction spherical equivalent
CCT: Central corneal thickness
LT: Lenticule thickness
RST: Residual stromal bed thickness
