# Supplementary material for: Corneal biomechanical characteristics following small incision lenticule extraction for myopia and astigmatism with 3 different cap thicknesses
Source: BMC Ophthalmol. 2023 Jan 30;23:42. doi: 10.1186/s12886-023-02786-8 (PMC9885585; doi:10.1186/s12886-023-02786-8)
Supplement: Supplementary file 1 — Additional file 1. Corneal Biomechanical Parameters at Different Time Points in Three Groups. [file 12886_2023_2786_MOESM1_ESM.docx]

| Additional file 1 Corneal Biomechanical Parameters at Different Time Points in Three Groups | | | | | | | |
| --- | --- | --- | --- | --- | --- | --- | --- |
| Parameter | Group | Preop | 1-Week Postop | 1-Month Postop | 3-Month Postop | 6-Month Postop | P Value |
| M (mm^-1^) | 110 μm | 0.18 ± 0.02 | 0.22 ± 0.02 | 0.22 ± 0.02 | 0.22 ± 0.01 | 0.22 ± 0.02 | <0.001* |
|  | 120 μm | 0.18 ± 0.02 | 0.22 ± 0.02 | 0.22 ± 0.02 | 0.22 ± 0.01 | 0.23 ± 0.05 | <0.001* |
|  | 130 μm | 0.18 ± 0.01 | 0.22 ± 0.01 | 0.22 ± 0.01 | 0.22 ± 0.03 | 0.22 ± 0.01 | <0.001* |
|  | P Value | 0.041* | 0.980 | 0.792 | 0.232 | 0.742 |  |
| IR (mm^-1^) | 110 μm | 8.86 ± 1.07 | 11.51 ± 1.04 | 11.54 ± 1.09 | 11.60 ± 0.89 | 11.83 ± 0.84 | <0.001* |
|  | 120 μm | 8.67 ± 0.80 | 11.63 ± 0.90 | 11.94 ± 1.05 | 11.83 ± 0.90 | 11.84 ± 0.83 | <0.001* |
|  | 130 μm | 8.41 ± 0.60 | 11.39 ± 0.72 | 11.59 ± 0.77 | 11.38 ± 0.61 | 11.31 ± 0.58 | <0.001* |
|  | P Value | 0.121 | 0.141 | 0.019* | 0.625 | 0.087 |  |
| DA ratio 1mm | 110 μm | 1.55 ± 0.04 | 1.67 ± 0.07 | 1.66 ± 0.05 | 1.66 ± 0.04 | 1.66 ± 0.04 | <0.001* |
|  | 120 μm | 1.54 ± 0.04 | 1.66 ± 0.03 | 1.67 ± 0.03 | 1.66 ± 0.03 | 1.65 ± 0.03 | <0.001* |
|  | 130 μm | 1.52 ± 0.03 | 1.66 ± 0.04 | 1.66 ± 0.03 | 1.66 ± 0.04 | 1.66 ± 0.04 | <0.001* |
|  | P Value | 0.072 | 0.900 | 0.310 | 0.072 | 0.752 |  |
| DA ratio 2mm | 110 μm | 4.19 ± 0.35 | 5.34 ± 0.48 | 5.40 ± 0.54 | 5.40 ± 0.42 | 5.55 ± 0.40 | <0.001* |
|  | 120 μm | 4.11 ± 0.29 | 5.35 ± 0.38 | 5.60 ± 0.31 | 5.52 ± 0.35 | 5.56 ± 0.66 | <0.001* |
|  | 130 μm | 3.99 ± 0.25 | 5.34 ± 0.46 | 5.51 ± 0.43 | 5.36 ± 0.35 | 5.33 ± 0.39 | <0.001* |
|  | P Value | 0.040* | 0.074 | 0.002* | 0.226 | 0.705 |  |
| ARTh | 110 μm | 453.76 ± 83.95 | 151.53 ± 30.08 | 150.89 ± 27.08 | 152.59 ± 19.47 | 153.60 ± 20.93 | <0.001* |
|  | 120 μm | 550.51 ± 126.44 | 157.99 ± 21.63 | 155.17 ± 21.81 | 164.00 ± 22.10 | 166.56 ± 22.21 | <0.001* |
|  | 130 μm | 484.30 ± 83.63 | 161.96 ± 23.71 | 161.57 ± 21.74 | 163.57 ± 19.53 | 168.28 ± 20.26 | <0.001* |
|  | P Value | 0.001* | 0.154 | 0.112 | 0.054 | 0.011* |  |
| SP-A1 | 110 μm | 107.43 ± 15.08 | 75.79 ± 18.72 | 68.41 ± 17.77 | 65.12 ± 11.77 | 65.85 ± 12.16 | <0.001* |
|  | 120 μm | 114.17 ± 11.82 | 75.11 ± 13.42 | 65.00 ± 12.48 | 67.66 ± 15.21 | 66.60 ± 11.65 | <0.001* |
|  | 130 μm | 109.19 ± 11.88 | 76.50 ± 14.15 | 69.98 ± 12.07 | 76.29 ± 11.05 | 77.36 ± 12.09 | <0.001* |
|  | P Value | 0.358 | 0.929 | 0.822 | <0.001* | <0.001* |  |
| SSI | 110 μm | 0.91 ± 0.14 | 0.87 ± 0.16 | 0.86 ± 0.22 | 0.79 ± 0.10 | 0.79 ± 0.10 | <0.001* |
|  | 120 μm | 0.91 ± 0.12 | 0.86 ± 0.14 | 0.79 ± 0.09 | 0.80 ± 0.11 | 0.81 ± 0.09 | <0.001* |
|  | 130 μm | 0.95 ± 0.11 | 0.87 ± 0.10 | 0.82 ± 0.09 | 0.85 ± 0.09 | 0.87 ± 0.09 | <0.001* |
|  | P Value | 0.494 | 0.241 | 0.055 | 0.030* | 0.002* |  |
| bIOP (mmHg) | 110 μm | 15.00 ± 1.95 | 13.69 ± 2.08 | 12.98 ± 2.61 | 12.20 ± 1.71 | 12.35 ± 1.67 | <0.001* |
|  | 120 μm | 15.08 ± 1.64 | 13.60 ± 1.88 | 12.14 ± 1.97 | 12.22 ± 2.07 | 12.44 ± 1.50 | <0.001* |
|  | 130 μm | 15.29 ± 1.70 | 13.50 ± 2.01 | 12.40 ± 1.47 | 13.17 ± 1.53 | 13.35 ± 1.80 | <0.001* |
|  | P Value | 0.496 | 0.288 | 0.078 | 0.027* | 0.023* |  |
| CCT (μm) | 110 μm | 547.34 ± 15.21 | 442.38 ± 26.26 | 441.12 ± 18.03 | 445.99 ± 16.12 | 445.23 ± 16.16 | <0.001* |
|  | 120 μm | 556.68 ± 18.60 | 441.75 ± 12.19 | 442.41 ± 12.32 | 448.94 ± 13.65 | 447.97 ± 14.32 | <0.001* |
|  | 130 μm | 553.24 ± 10.96 | 450.66 ± 11.25 | 450.42 ± 12.62 | 456.82 ± 11.81 | 457.95 ± 9.97 | <0.001* |
|  | P Value | 0.492 | 0.159 | 0.037* | 0.008* | 0.001* |  |
| CBI-LVC | 110 μm |  | 0.000 (0.000, 0.000) | 0.000 (0.000, 0.000) | 0.000 (0.000, 0.000) | 0.000 (0.000, 0.000) | 0.790 |
|  | 120 μm |  | 0.000 (0.000, 0.000) | 0.000 (0.000, 0.001) | 0.000 (0.000, 0.000) | 0.000 (0.000, 0.000) | 0.679 |
|  | 130 μm |  | 0.000 (0.000, 0.000) | 0.000 (0.000, 0.001) | 0.000 (0.000, 0.000) | 0.000 (0.000, 0.000) | 0.195 |
|  | P Value |  | 0.590 | 0.244 | 0.990 | 0.096 |  |

M, max inverse radius; IR, integrated radius; DA ratio, deformation amplitude ratio; ARTh, Ambrósio relational thickness; SP-A1, stiffness parameter at applanation A1; SSI, stress-strain index; bIOP, biomechanically corrected intraocular pressure; CCT, central corneal thickness; CBI-LVC, Corvis biomechanical index-laser vision correction.

*P < 0.05.
